# Significance of Self‐Expandable Metallic Stent for Postoperative Intra‐Abdominal Infection After Pancreatoduodenectomy in Patients With a Hard Pancreas

**DOI:** 10.1002/ags3.70150

**Published:** 2025-12-15

**Authors:** Kosuke Mori, Haruyoshi Tanaka, Nana Kimura, Mina Fukasawa, Ayaka Itoh, Yoshihiro Shirai, Kazuto Shibuya, Kentaro Nagaoka, Yoshihiro Yamamoto, Tsutomu Fujii

**Affiliations:** ^1^ Department of Surgery and Science, Faculty of Medicine, Academic Assembly University of Toyama Toyama Japan; ^2^ Department of Surgery Nagoya University Hospital Nagoya Aichi Japan; ^3^ Clinical and Research Center for Infectious Diseases Toyama University Hospital Toyama Japan

**Keywords:** bacterial culture, biliary drainage, intra‐abdominal infection, pancreatoduodenectomy, self‐expandable metal stent

## Abstract

**Background:**

Self‐expandable metal stents (SEMS) are often used for preoperative biliary drainage in pancreatoduodenectomy (PD); however, their impact on postoperative intra‐abdominal infection (POAI) remains unclear. This study aimed to evaluate the clinical significance of SEMS in relation to POAI.

**Methods:**

The data of 314 consecutive patients who underwent elective PD between January 2018 and May 2022 were retrospectively analyzed. Patients were categorized by biliary drainage method (NON: no preoperative biliary drainage, PS: plastic stent, or SEMS), and the associations with POAI were assessed. Bile and drainage fluid cultures were microbiologically examined.

**Results:**

Soft pancreatic texture and preoperative biliary drainage were independently associated with POAI. In the hard pancreas subgroup undergoing preoperative biliary drainage (*n* = 45), all POAI cases occurred despite low drain fluid amylase levels and the absence of clinically relevant postoperative pancreatic fistula (POPF). SEMS placement was the only significant risk factor for POAI (odds ratio 4.38 [1.21–18.73], *p* = 0.03). The concordance rate between organisms in bile and drainage fluid was 66.7% in the SEMS group, 52.2% in the PS group, and 7.2% in the NON group. In the SEMS group, *E. cloacae* , *E. faecalis* , and *E. faecium* were the most frequently isolated organisms (38.1%, 33.3%, and 19.0%, respectively).

**Conclusion:**

SEMS placement was associated with an increased risk of POAI in patients with a hard pancreas, who are unlikely to develop POPF. Bile cultures may assist in predicting the causative organisms of POAI in patients who undergo preoperative biliary drainage.

## Graphical abstract

In patients undergoing pancreatoduodenectomy, preoperative SEMS placement was associated with a significantly higher risk of postoperative intra‐abdominal infection (POAI), even in those with a hard pancreas and without clinically relevant POPF. Bile and drainage cultures frequently matched in the SEMS group, suggesting that bile contamination contributes to infection. Bile cultures may help predict causative organisms in patients receiving preoperative biliary stents.

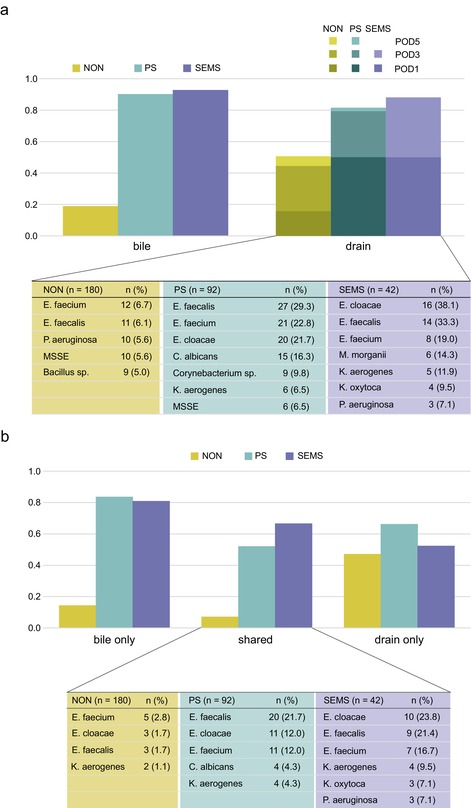

## 1 Introduction

Surgical site infection remains a major postoperative complication following pancreatoduodenectomy (PD), contributing to delayed recovery and impaired initiation of adjuvant therapy. In particular, postoperative intra‐abdominal infections (POAI), such as abscesses or infected fluid collection in the peritoneal cavity, can lead to severe morbidity, including organ failure [1, 2, 3, 4].

While most cases of POAI after PD are attributable to postoperative pancreatic fistula (POPF), another important contributing factor is intraoperative contamination of the peritoneal cavity [5, 6]. Despite a hard pancreatic texture, which usually indicates a low risk of POPF and often leads to early drain removal, some patients develop late‐onset POAI, partly due to an increased bacterial load in the peritoneal cavity [5, 7]. Considering that bacterial contamination plays a crucial role in the development of POAI, preoperative biliary drainage represents an important source of bacterial contamination [1, 8, 9, 10, 11, 12, 13, 14].

Nevertheless, biliary drainage is necessary in patients with severe obstructive jaundice or biliary tract infection. In particular, self‐expandable metal stents (SEMSs) offer the advantage of long‐term patency, which is beneficial for patients undergoing longer preoperative treatment, and their use has increased recently. However, the impact of SEMSs on POAI has not been well studied, and the bacterial landscape associated with different biliary drainage systems remains poorly characterized. Therefore, this study aimed to evaluate the clinical impact of SEMSs on POAI, with particular attention to biliary drainage as a potential source of bacterial contamination.

## 2 Methods

### 2.1 Study Design

This retrospective study included 314 consecutive patients who underwent elective PD with pancreatojejunostomy at Toyama University Hospital between January 2018 and May 2022. Patients were classified into three groups based on the type of preoperative biliary drainage: no drainage (NON group), plastic stent (PS group), and self‐expandable metal stent (SEMS group), according to the principal drainage method when multiple procedures were performed. Due to the observational nature of this study, no predefined sample size calculation or specific intervention was applied.

### 2.2 Surgical Techniques and Perioperative Management

Preoperative biliary drainage was indicated for patients with jaundice. In most cases, stent selection was not standardized and was determined on a case‐by‐case basis. A 10 mm SEMS, a 7 Fr PS, or a 6 Fr endoscopic nasobiliary drainage tube was selected depending on the clinical context. Specifically, a SEMS was increasingly chosen for patients with pancreatic cancer, who often required a longer period of preoperative chemotherapy, whereas a PS was more frequently used as an initial drainage option for patients with jaundice, cholangitis, or cholangiocarcinoma, sometimes to avoid surgical difficulty caused by periductal fibrosis.

Pancreatic texture was classified as soft or hard based on intraoperative palpation by the operating surgeon, and the classification was recorded in the operative notes. Pancreatojejunostomy was generally performed using the modified Blumgart technique [15]. Pancreatic duct diameter was measured intraoperatively at the pancreatic cut surface using a sterile surgical ruler, immediately after placement of eight ductal stitches during the modified Blumgart procedure, with confirmation by the operating surgeon and assistant. Three closed‐suction drains were placed: two around the pancreatojejunostomy and one near the choledochojejunostomy. Intraoperative bile and postoperative drainage fluid were routinely cultured on postoperative days (PODs) 1, 3, and 5, and postoperative antibiotics were changed as necessary according to the culture results. The drains were usually removed between POD3 and POD5 when the fluid was clear and the amylase level was < 3 times the upper normal serum limit, in accordance with International Study Group of Pancreatic Surgery (ISGPS) 2016 criteria. Computed tomography (CT) was routinely performed on POD4, and drains were exchanged over a guidewire or additional percutaneous drainage was performed as needed.

### 2.3 Data Collection

Clinical data potentially associated with postoperative outcomes were retrospectively collected from electronic medical records. POAI was defined as either (1) intra‐abdominal infection of Clavien–Dindo Grade II or higher, or (2) clinically relevant POPF of Grade B or C, based on the 2016 ISGPS classification [16, 17].

Intra‐abdominal infection was diagnosed based on the presence of clinical symptoms, such as fever; radiologic or ultrasonographic evidence of fluid collection or abscess; microbiological confirmation by a positive drainage fluid culture; and the absence of other organ infections, such as cholangitis. Mortality was defined as in‐hospital death within 30 days after surgery.

### 2.4 Statistical Analysis

Descriptive statistics, including means, standard deviations, medians, and proportions, are used to summarize demographic and clinical characteristics. The primary outcome was the occurrence of POAI. Fisher's exact test was used to analyze categorical variables, and the Mann–Whitney U test for continuous variables. Univariate and multivariate logistic regression analyses were performed. Variables that were statistically significant in univariate analysis were entered into the multivariate model. All analyses were performed using R version 4.2.3 (R Foundation for Statistical Computing, Vienna, Austria), and *p* < 0.05 was considered statistically significant.

### 2.5 Ethics

This study was approved by the Ethics Committee of Toyama University Hospital (Approval No. R2022192) and was conducted in accordance with the Declaration of Helsinki and the principles of human rights protection.

## 3 Results

The baseline characteristics of 314 patients are summarized in Table 1. The mean age was 70.9 ± 10.7 years. Primary diagnoses included 122 cases of pancreatic cancer, 80 of bile duct cancer, and 68 of intraductal papillary mucinous neoplasms (IPMNs). Preoperative biliary drainage was performed in 134 patients. The overall median duration of preoperative biliary drainage was 75.5 days (IQR, 36.5–111). By drainage type, the median duration of preoperative biliary drainage was 58 days (IQR, 31.3–98) in the PS group and 105 days (IQR, 72.8–232) in the SEMS group. Thirty‐six patients experienced 63 episodes of unplanned stent obstruction or malfunction requiring replacement, ranging from two to seven per patient. Including routine or prophylactic exchanges, most patients undergoing biliary drainage received at least one stent replacement before surgery, with the total number of exchanges ranging from zero to seven. Open surgery was performed in 304 (96.8%) patients, laparoscopic surgery in 3 (1.0%), and robot‐assisted surgery in 7 (2.2%). Pancreatic texture was classified as hard in 105 patients and soft in 207; data were unavailable for two patients. POAI was more frequent in patients who underwent preoperative drainage. Correspondingly, the resistance rates of microorganisms isolated from postoperative drain cultures to prophylactic antibiotics on POD1 were 17.8%, 75.0%, and 90.5% in the NON, PS, and SEMS groups, respectively. In particular, among patients without POPF in the SEMS group, 30/34 (88.2%) had positive drain cultures. POAI occurred in 17/30 (56.7%) culture‐positive patients, whereas none occurred in culture‐negative patients.

**TABLE 1:** Baseline characteristics and operative outcomes of the entire cohort.

Variables	Overall	NON	PS	SEMS	*p*
	*N* = 314	*n* = 180	*n* = 92	*n* = 42	
Age (mean (SD))	70.9 (10.7)	69.7 (12.1)	72.8 (8.2)	72.1 (7.8)	0.061
Female/Male (*n* (%))	123/191 (39.2/60.8)	71/109 (39.4/60.6)	37/55 (40.2/59.8)	15/27 (35.7/64.3)	0.88
ASA–PS (*n* (%))					0.19
I	33 (10.5)	18 (10.0)	12 (13.0)	3 (7.1)	
II	135 (43.0)	78 (43.3)	34 (37.0)	23 (54.8)	
III	144 (45.9)	84 (46.7)	44 (47.8)	16 (38.1)	
IV	2 (0.6)	0 (0.0)	2 (2.2)	0 (0.0)	
BMI (mean (SD))	22.4 (3.4)	22.8 (3.6)	22.1 (3.1)	20.9 (2.9)	0.003
PNI (mean (SD))	37.7 (4.9)	39.5 (3.8)	35.0 (4.6)	35.6 (6.1)	< 0.001
Primary disease (*n* (%))					< 0.001
Pancreatic cancer	148 (47.1)	91 (50.6)	26 (28.3)	31 (73.8)	
Bile duct cancer	80 (25.5)	10 (5.6)	59 (64.1)	11 (26.2)	
IPMN	68 (21.7)	63 (35.0)	5 (5.4)	0 (0.0)	
Others	18 (5.7)	16 (8.9)	2 (2.2)	0 (0.0)	
Length of preoperative chemotherapy (median [IQR])	**0 [0–39.8]**	**0 [0–34.0]**	**0 [0–29.8]**	**53.5 [3.3–198]**	**< 0.001**
Preoperative biliary infection	**47 (15.0)**	**0 (0.0)**	**29 (31.5)**	**18 (42.9)**	**< 0.001**
Biliary drainage malfunction	**30 (9.6)**	**0 (0.0)**	**19 (20.7)**	**11 (26.2)**	**< 0.001**
Length of biliary drainage (median [IQR])	**75.5 [36.5–111]**	**NA**	**58.0 [31.3–98]**	**105 [72.8–232]**	**< 0.001**
Biliary drainage exchange number, (median[range])	**1 [0–7]**	**NA**	**1 [0–4]**	**1 [0–7]**	**0.106**
MIS approach (*n* (%))*	10 (3.2)	9 (5.0)	0 (0.0)	1 (2.4)	0.081
Operative time, min (mean (SD))	571.3 (153.5)	564.5 (165.5)	562.0 (128.0)	620.5 (145.4)	0.082
Blood loss, ml (mean (SD))	710.8 (551.8)	654.7 (571.5)	768.4 (569.2)	825.0 (380.2)	0.097
Vessel resection (*n* (%))					
Portal vein	75 (23.9)	40 (22.2)	15 (16.3)	20 (47.6)	< 0.001
Artery	9 (2.9)	6 (3.3)	1 (1.1)	2 (4.8)	0.42
Pancreatic texture (*n* (%))					< 0.001
hard	104 (33.1)	59 (32.8)	18 (19.6)	27 (64.3)	
soft	208 (66.2)	120 (66.7)	73 (79.3)	15 (35.7)	
unknown	2 (0.6)	1 (0.6)	1 (1.1)	0 (0.0)	
Diameter of MPD, mm (median [IQR])*	**3.0 [2.0–5.0]**	**3.0 [2.0–5.0]**	**3.0 [2.4–4.0]**	**4.0 [3.0–5.0]**	**0.045**
Prophylactic antibiotics (*n* (%))					< 0.001
Cephazolin	227 (72.3)	143 (79.4)	56 (60.9)	28 (66.7)	
Cefmetazole	59 (18.8)	34 (18.9)	16 (17.4)	9 (20.5)	
Tazobactam/Piperacillin	5 (1.6)	0 (0)	5 (5.4)	0 (0)	
Cefepime	2 (0.6)	0 (0)	2 (2.2)	0 (0)	
Levofloxacin	2 (0.6)	0 (0)	2 (2.2)	0 (0)	
Others	19 (6.1)	3 (1.7)	11 (12.0)	5 (11.4)	
Covered by the prophylactics					**< 0.001**
Covered	**153 (48.7)**	**131 (72.8)**	**18 (19.6)**	**4 (9.5)**	
Not covered	**139 (44.3)**	**32 (17.8)**	**69 (75.0)**	**38 (90.5)**	
Not available	**22 (7.0)**	**17 (9.4)**	**5 (5.4)**	**0 (0.0)**	
AMY‐D at POD3, IU/ml (median [IQR])	541.5 [85.3–1898.3]	576.5 [84.0–1876.8]	860.5 [237.3–2772.8]	96.0 [32.8–488.5]	< 0.001
POPF, ISGPS 2016 (*n* (%))					0.047
none	151 (48.1)	87 (48.3)	36 (39.1)	28 (66.7)	
BL	70 (22.3)	44 (24.4)	20 (21.7)	6 (14.3)	
B	92 (29.3)	49 (27.2)	35 (38.0)	8 (19.0)	
C	1 (0.3)	0 (0.0)	1 (1.1)	0 (0.0)	
POAI	**172 (54.8)**	**88 (48.9)**	**59 (64.1)**	**25 (59.5)**	**0.06**
Bile leakage	**11 (3.5)**	**9 (5.0)**	**1 (1.1)**	**1 (2.4)**	**0.23**
Morbidity and Mortality C–D (*n*(%))^†^					0.21
none	121 (38.5)	72 (40.0)	31 (33.7)	18 (42.9)	
I	7 (2.2)	3 (1.7)	4 (4.3)	0 (0.0)	
II	126 (40.1)	76 (42.2)	35 (38.0)	15 (35.7)	
IIIa	53 (16.9)	26 (14.4)	20 (21.7)	7 (16.7)	
IIIb	5 (1.6)	3 (1.7)	1 (1.1)	1 (2.4)	
IVb	1 (0.3)	0 (0.0)	0 (0.0)	1 (2.4)	
V	1 (0.3)	0 (0.0)	1 (1.1)	0 (0.0)	

Abbreviations: AMY‐D, amylase in drainage fluid; ASA–PS, American Society of Anesthesiologists–Performance Status; BMI, Body mass index; IPMN, Intraductal papillary mucinous neoplasm; ISGPS, International surgical grading in pancreatic surgery; MIS, minimal invasive surgery; MPD, diameter of the main pancreatic duct; PNI, Prognostic nutritional index; POAI, postoperative abdominal infection; POD, postoperative day; POPF, postoperative pancreatic fistula; SSI, surgical site infection; C–D, Clavien–Dindo classification; T‐Bil, Total bilirubin.

* *n* = 312.

^†^ Within 30 postoperative days.

We investigated pre‐ and intraoperative risk factors for POAI in all 314 patients. Consistent with previous reports on POPF, soft pancreatic texture, preoperative biliary drainage, and higher body mass index were identified as independent risk factors for POAI [odds ratio (OR) 4.18, *p* < 0.001; OR 2.20, *p* = 0.003; OR 1.95, *p* = 0.039, respectively] (Table 2).

**TABLE 2:** Risk factors for postoperative abdominal infections (POAI) in all patients (*n* = 314).

Variables		POAI *n* (%)	Univariate		Multivariate	
			Odds ratio (95% CI)	*p*	Odds ratio (95% CI)	*p*
Age	≥ 70	103 (53.6)	0.98 (0.62–1.55)	0.94		
	< 70 (ref)	66 (54.1)				
Sex	male	104 (54.5)	1.07 (0.68–1.68)	0.78		
	female (ref)	65 (52.8)				
ASA–PS	III/IV	74 (51.4)	0.84 (0.53–1.30)	0.43		
	I – III (ref)	95 (55.9)				
BMI	≥ 25	41 (65.1)	1.79 (1.02–3.22)	0.047	**1.95 (1.04–3.74)**	**0.039**
	< 25 (ref)	128 (51)				
PNI	< 40	103 (53.1)	0.93 (0.59–1.46)	0.74		
	≥ 40 (ref)	66 (55)				
Biliary drainage	yes	83 (61.9)	1.78 (1.13–2.81)	0.013	**2.20 (1.32–3.73)**	**0.003**
	no (ref)	86 (47.8)				
Preoperative chemotherapy	yes	48 (43.2)	0.51 (0.32–0.82)	0.006	0.89 (0.51–1.61)	0.71
	no (ref)	121 (59.6)				
Operative approach	MIS	6 (60)	1.30 (0.36–5.16)	0.69		
	open (ref)	163 (53.6)				
Diameter of MPD (mm)*	**≤ 3.0**	**106 (65.4)**	**2.76 (1.75–4.39)**	**< 0.001**	**1.59 (0.93–2.71)**	**0.085**
	**> 3.0 (ref)**	**61 (40.7)**				
Pancreatic texture*	soft	138 (66.3)	5.10 (3.07–8.65)	< 0.001	**4.18 (2.27–7.92)**	**< 0.001**
	hard (ref)	29 (27.9)				
Operative time (min)	≥ 600	70 (59.8)	1.47 (0.93–2.35)	0.10		
	< 600 (ref)	99 (50.3)				
Blood loss (ml)	≥ 500	101 (55.8)	1.21 (0.77–1.89)	0.41		
	< 500 (ref)	68 (51.1)				

Abbreviations: ASAPS, American Society of Anesthesiologists Performance Status; BMI, Body mass index; CI, confidence interval; MIS, minimally invasive surgery; MPD, diameter of the main pancreatic duct; PNI, prognostic nutritional index; POPF, Postoperative pancreatic fistula; SSI, surgical site infection.

* *n* = 312.

We next compared the impact of SEMS and PS placement on POAI in 45 patients who underwent preoperative biliary drainage and had a hard pancreas (18 in the PS group and 27 in the SEMS group). Table 3 shows the baseline characteristics of this subgroup. Drainage fluid amylase (DFA) levels on POD3 were generally low, and no POAI cases overlapped with clinically relevant POPF. In this subgroup, SEMS placement was identified as a significant risk factor for POAI (OR 4.38, *p* = 0.032) (Table 4).

**TABLE 3:** Characteristics and operative outcome between the SEMS and PS groups among patients with hard pancreas and internal drainage.

Variables	PS	SEMS	*p*
	*n* = 18	*n* = 27	
Age (mean (SD))	68.8 (12.9)	70.56 (8.2)	0.57
Female/Male (*n* (%))	11/7 (61.1/38.9)	12/15 (44.4/55.6)	0.43
ASA‐PS (*n* (%))			0.909
I	1 (5.6)	1 (3.7)	
II	10 (55.6)	14 (51.9)	
III	7 (38.9)	12 (44.4)	
BMI (mean (SD))	22.1 (2.9)	20.3 (2.8)	**0.048**
PNI (mean (SD))	34.0 (5.5)	35.3 (7.0)	0.51
Primary disease (*n* (%))			0.072
pancreatic cancer	12 (66.7)	25 (92.6)	
bile duct cancer	5 (27.8)	2 (7.4)	
IPMN	1 (5.6)	0 (0.0)	
Preoperative chemotherapy (*n* (%))	11 (61.1)	25 (92.6)	**0.027**
Operative time, min (mean (SD))	583.0 (143.8)	646.9 (158.1)	0.18
Blood loss, ml (mean (SD))	931.9 (973.5)	915.0 (424.5)	0.94
Portal vein resection (*n* (%))	7 (38.9)	17 (63.0)	0.20
Arterial resection (*n* (%))	0 (0)	1 (3.7)	1
MPD, mm (median [IQR])	**4.75 [4.0–5.0]**	**5.0 [3.5–5.0]**	**0.924**
AMY‐D at POD3 (median [IQR])	79.0 [44.8–142.8]	42.0 [20.5–86.0]	**0.032**
POPF, ISGPS 2016 (*n* (%))			1
None	16 (88.9)	25 (92.6)	
BL	2 (11.1)	2 (7.4)	
Bile leakage (*n* (%))	**0 (0)**	**1 (3.7)**	**1**
Morbidity and Mortality C–D (*n* (%))^†^			0.129
None	8 (44.4)	11 (40.7)	
I	2 (11.1)	0 (0.0)	
II	7 (38.9)	10 (37.0)	
IIIa	0 (0.0)	5 (18.5)	
IIIb	0 (0.0)	1 (3.7)	
V	1 (5.6)	0 (0.0)	

Abbreviations: AMY‐D, amylase in drainage fluid; ASA‐PS, American Society of Anesthesiologists‐Physical Status; BMI, body mass index; C–D, Clavien–Dindo classification; IPMN, intraductal papillary mucinous neoplasm; IQR, inter quartile range; ISGPS, International Study Group in Pancreatic Surgery; MPD, main pancreatic duct; POD, postoperative day; POPF, postoperative pancreatic fistula; PS, plastic stent; SD, standard deviation; SEMS, self‐expandable metal stent; SSI, surgical site infection.

^†^ Within 30 postoperative days.

**TABLE 4:** Univariate analysis for pre‐ and intra‐operative risk factors for POAI in patients with hard pancreas and internal drainage.

Variables		POAI *n* (%)	Univariate	
			Odds ratio (95% CI)	*p*
Age	≥ 70	12 (48)	1.71 (0.52–5.94)	0.38
	< 70 (ref)	7 (35)		
Sex	Male	10 (45.5)	1.296 (0.40–4.31)	0.67
	Female (ref)	9 (39.1)		
ASA‐PS	≥ III	9 (47.4)	1.44 (0.43–4.85)	0.55
	< III (ref)	10 (38.5)		
BMI	≥ 25	2 (40)	0.90 (0.11–6.03)	0.92
	< 25 (ref)	17 (42.5)		
PNI	< 40	14 (40)	0.67 (0.16–2.81)	0.57
	≥ 40 (ref)	5 (50)		
Biliary drainage	SEMS	15 (55.6)	**4.38 (1.21–18.7)**	**0.032**
	PS (ref)	4 (22.2)		
Preoperative chemotherapy	yes	16 (44.4)	1.60 (0.36–8.53)	0.548
	no (ref)	3 (33.3)		
Diameter of MPD (mm)	≤ 3	6 (60)	2.54 (0.61–11.6)	0.21
	> 3 (ref)	13 (37.1)		
Coverage of bacteria from pre/intra operative biliary drainage	Not covered	**18 (43.9)**	**2.35 (0.27–49.7)**	**0.48**
	Covered (ref)	**1 (25)**		
Operative time (min)	≥ 600	15 (53.6)	3.75 (1.04–16.0)	0.054
	< 600 (ref)	4 (23.5)		
Blood loss (ml)	≥ 500	16 (44.4)	1.6 (0.36–8.53)	0.55
	< 500 (ref)	3 (33.3)		

Abbreviations: ASA‐PS, American Society of Anesthesiologists physical status; BMI, body mass index; CI, confidence interval; MPD, main pancreatic duct; PNI, prognostic nutritional index; POAI, postoperative abdominal infection; PS, plastic stent; SEMS, self‐expandable metal stent.

**FIGURE 1:**
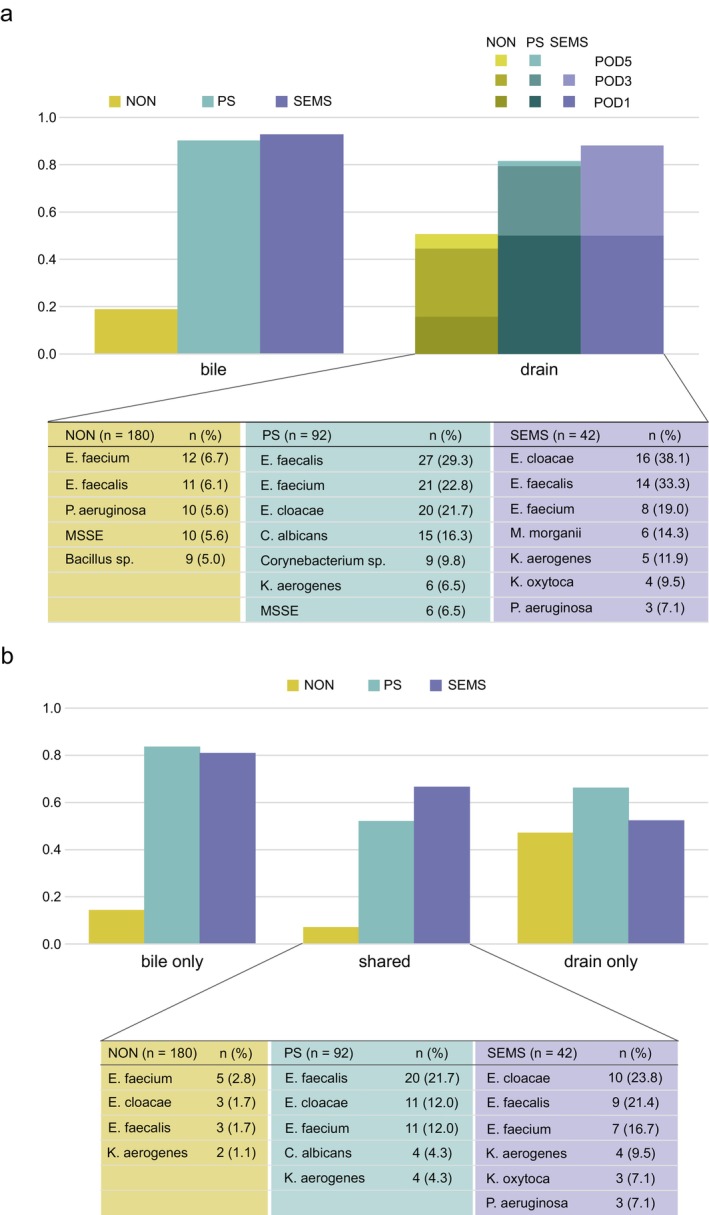
Bacterial profiling results. Many bacteria detected in drainage fluid were consistent with bile juice culture in biliary drainage cases. (a) Bacterial species detected in bile juice (left panel) and drainage fluid (right panel) according to each preoperative biliary drainage type, and rates of samples from which day causative bacteria were initially isolated. Refer to Table S2 for a list of isolated bacteria from bile and drainage fluid, respectively. (b) Bacterial species detected only in bile (right panel) and drainage fluid (left panel), and shared in both sources (middle). Please refer to the full list of shared bacteria in Table S3 for the no biliary drainage (NON) group, Table S4 for the plastic stent (PS) group, and Table S5 for the self‐expandable metal stents (SEMS) group. MSSE, methicillin‐susceptible *S. epidermidis*.

We also profiled the bacteria identified in drainage and bile cultures. Positive culture rates were higher in the biliary drainage groups (PS and SEMS) than in the NON group. The highest positive culture rate was observed on POD1, followed by POD3, in both the PS and SEMS groups, whereas the NON group showed a lower proportion on POD1 (Figure 1a). *Enterococcus faecalis* , *Enterococcus faecium* , and *Enterobacter cloacae* were the most frequently identified organisms in all groups, whereas anaerobes such as *Bacteroides* and *Clostridium* were detected in fewer than 3% of the cases (Table S1). The bacterial profile of the drainage fluid more closely reflected that of the bile in the biliary drainage groups, particularly in the SEMS group (66.7%) (Figure 1b). *E. cloacae* was the most common organism in the SEMS group (23.8%). *Pseudomonas aeruginosa* was more frequently isolated in the SEMS group than in the other groups (Figure 1b, Table S5).

## 4 Discussion

POAI remains challenging to predict and control after PD in cases without overt pancreatic fistula [6, 18]. In the present study, a small but clinically relevant subset of patients with hard pancreas who underwent preoperative biliary drainage developed POAI in the absence of POPF. Most patients required additional antimicrobial therapy or interventional drainage. It has been our policy to remove drains on POD4 following routine CT evaluation, provided that DFA levels are low. In SEMS cases, delaying drain removal, waiting for final drain fluid culture results, and considering drain exchange as needed may be beneficial. This approach allows time to consider antibiotic selection based on culture results, rather than removing the drain prematurely. Beyond postoperative management, additional intraoperative measures, such as peritoneal lavage before closure and careful suctioning during biliary reconstruction, may further help reduce bacterial contamination in SEMS cases.

Pre‐ or intra‐operative bile cultures showed reasonable concordance with postoperative pathogens, especially in biliary drainage groups. These findings suggest that such cultures may help guide perioperative antibiotic selection in patients undergoing biliary drainage, whereas their utility appears limited in those without. The bacterial landscape depicted in this cohort further highlighted issues relevant to postoperative antimicrobial management. In all groups, *E. faecalis* , *E. faecium* , and *E. cloacae* were the most frequently isolated pathogens from drainage fluid. Notably, *E. faecium* was isolated at a similar frequency to *E. faecalis* , whereas *E. faecalis* typically predominates in the normal intestinal flora. This finding suggests a shift toward hospital‐associated species, possibly influenced by prior antibiotic exposure and preoperative invasive procedures [19, 20]. This shift may complicate postoperative management, because unlike *E. faecalis* , *E. faecium* is intrinsically resistant to penicillin and cephalosporins. In the SEMS group, *E. cloacae* and *P. aeruginosa* , representative drug‐resistant gram‐negative rods, were more frequently detected than in the other groups. Immunosuppression associated with malignancy and the relatively longer duration of preoperative chemotherapy may have contributed to this bacterial profile. Greater attention may therefore be warranted when selecting perioperative antibiotics in SEMS cases, with consideration for coverage against resistant organisms.

Most of the cultured bacteria were aerobic. Although some studies support the use of anaerobic coverage in intra‐abdominal infections, prophylactic cefazolin remains the guideline‐recommended antibiotic for elective PD [21, 22]. The necessity of anaerobic coverage remains uncertain in surgically drained infections, in which anaerobes are infrequently detected. Empirical anaerobic coverage using β‐lactam/β‐lactamase inhibitors (e.g., piperacillin–tazobactam) may be discouraged due to the risk of inducing AmpC β‐lactamase expression, which could complicate the management of future infections [23, 24].

This study has certain limitations. First, its retrospective single‐center design and relatively small sample size may limit the generalizability of the findings, particularly for subgroup analyses in patients with a hard pancreas. A prospective, multicenter study with a larger cohort is warranted to validate these results. Second, the selection of stent type was neither standardized nor randomized, potentially introducing selection bias based on tumor characteristics and planned treatment duration. This study does not discourage the use of SEMS for biliary drainage but underscores the importance of minimizing intraoperative contamination from bile. Early drain removal should be approached with caution until microbiological culture results are available, especially in patients with SEMS.

In conclusion, preoperative biliary drainage, particularly using SEMS, was associated with an increased risk of POAI in patients with a hard pancreas, who are unlikely to develop POPF. Unlike in drainage‐naïve patients, bile cultures may assist in predicting the causative organisms of POAI in those who undergo preoperative biliary drainage.

## Author Contributions

**Kosuke Mori:** writing – original draft, data curation, methodology, formal analysis, visualization, validation. **Haruyoshi Tanaka:** conceptualization, methodology, data curation, investigation, formal analysis, visualization, project administration, writing – original draft, software, funding acquisition, writing – review and editing, resources. **Nana Kimura:** writing – review and editing, validation, data curation. **Mina Fukasawa:** validation, writing – review and editing, data curation, resources. **Ayaka Itoh:** validation, writing – review and editing, data curation. **Yoshihiro Shirai:** validation, writing – review and editing, data curation, project administration, resources. **Kazuto Shibuya:** validation, writing – review and editing, data curation, resources. **Kentaro Nagaoka:** resources, writing – review and editing, investigation. **Yoshihiro Yamamoto:** resources, writing – review and editing, investigation, funding acquisition. **Tsutomu Fujii:** supervision, project administration, writing – review and editing, funding acquisition, conceptualization.

## Funding

This work was supported by Japan Society for the Promotion of Science (JSPS), (JP23K08209).

## Ethics Statement

The protocol for this research project has been approved by a suitably constituted Ethics Committee of the institution, and it conforms to the provisions of the Declaration of Helsinki. Committee of Toyama University Hospital, Approval No. R2022192. Informed consent was obtained from all the subjects.

## Consent

Informed consent was obtained from all patients.

## Conflicts of Interest

Dr. Tsutomu Fujii is an editorial board member of Annals of Gastroenterological Surgery. All other authors declare no conflicts of interest.

## Supporting information


**Table S1:** Bacterial species isolated from intra‐ or preoperative bile in all patients.

**Table S2:** Bacterial species isolated from postoperative drainage fluid in all patients.

**Table S3:** Bacterial species shared between bile juice and drainage fluid in patients not undergoing biliary drainage (NON group).

**Table S4:** Bacterial species shared between bile juice and drainage fluid in patients receiving plastic stents (PS group).

**Table S5:** Bacterial species shared between bile juice and drainage fluid in patients receiving self‐expandable metallic stents (SEMS group).

## Data Availability

The data supporting the findings of this study are available upon reasonable request to the corresponding author.
